# Carbon Nanotubes-Sponge Modified Electro Membrane Bioreactor (EMBR) and Their Prospects for Wastewater Treatment Applications

**DOI:** 10.3390/membranes10120433

**Published:** 2020-12-17

**Authors:** Ali M. Almusawy, Riyad H. Al-Anbari, Qusay F. Alsalhy, Arshed Imad Al-Najar

**Affiliations:** 1 Civil Engineering Department, University of Technology, Alsinaa Street 52, Baghdad 10066, Iraq; 40393@student.uotechnology.edu.iq (A.M.A.); riyadhassan2003@yahoo.com (R.H.A.-A.); aarshad_emad@yahoo.com (A.I.A.-N.); 2 Membrane Technology Research Unit, Chemical Engineering Department, University of Technology, Alsinaa Street 52, Baghdad 10066, Iraq

**Keywords:** membrane bioreactor, electrochemistry, carbon nanotube, membrane fouling, wastewater

## Abstract

A novel membrane bioreactor system utilizes Multi-Walled Carbon Nanotubes (MWCNTs) coated polyurethane sponge (PUs), an electrical field, and a nanocomposite membrane has been successfully designed to diminish membrane with fouling caused by activated sludge. The classical phase inversion was harnessed to prepare Zinc Oxide/Polyphenylsulfone (ZnO/PPSU) nanocomposite membranes using 1.5 g of ZnO nanoparticles (NPs). The prepared nanocomposite membrane surface was fully characterized by a series of experimental tools, e.g., Scanning electron microscope (SEM), Atomic force microscopy (AFM), contact angle (CA), pore size, and pore size distribution. The testing procedure was performed through an Activated Sludge-Membrane Bioreactor (ASMBR) as a reference and results were compared with those obtained with nanotubes coated sponge–MBR (NSMBR) and nanotubes coated sponge-MBR in the presence of an electrical field (ENSMBR) system. Observed fouling reduction of the membrane has improved significantly and, thus, the overall long-term was increased by 190% compared with the control ASMBR configuration. The experimental results showcased that sponge-carbon nanotubes (CNTs) were capable of adsorbing activated sludge and other contaminants to minimize the membrane fouling. At a dosage of 0.3 mg/mL CNT and 2 mg/mL of SDBS, the sponge-CNT was capable of eliminating nitrogen and phosphorus by 81% and >90%, respectively.

## 1. Introduction

The rapid growth, witnessed in recent decades in industrialization and population, has given rise to release massive organic contamination into the aquatic system [1,2]. Versatile treatment technologies have been harnessed to cope with the endless catastrophic consequences occasioned by the presence of these organic components. Advanced oxidation (e.g., ozonation and UV-photocatalysis) and membrane filtration processes are ubiquitous examples of these techniques. Despite the relatively advanced state of these technologies, there is a huge debate about which one can outweigh among the other. In this context, membrane bioreactor (MBR) systems have emerged as a potent alternative to the traditional biological processes for the reclamation and removal of nutrients and carbon sources [3,4,5,6]. The leading characteristics of MBR are represented by the low sludge generation, typical retention, and exemplary separation efficiency with minimal space requirements. However, like any other membrane separation process, external and internal membrane fouling is an inevitable phenomenon associated with MBR systems. Fouling topics have been covered by an enormous research activity since the first membrane’s inception. Among the available methods, process modification through nanomaterials has offered an unlimited research area to diminish fouling with the aid of electrochemistry.

Among the wide diversity of available nanostructures, the carbon-based nanomaterials, e.g., one-dimensional carbon nanotubes (CNTs), have attracted the scientific community owing to their optical and mechanical characteristics, tunable electrical properties, light weight, and high specific area [7]. In the water treatment industry, electrochemistry has been involved in oxidizing the pollutants in an aqueous form. These outstanding characteristics have been employed for the manufacturing of highly porous networks for pollutants’ adsorption [8]. The way has been paved toward their application in electrodes manufacturing for adsorbing organic contaminants and degrading them through electrochemical oxidation [9]. The combination of electrochemistry with CNT filters has been reported as an efficient way for adsorptive trapping and electro-oxidant degradation of organic pollutants. [10] Claimed that CNT-based electric filter technology can effectively eliminate certain contaminants such as pharmaceuticals, colourants, viruses, bacteria, and organic aromatic compounds from aquatic environments.

Nevertheless, the high specific surface area/volume ratios of zero-dimensional nanomaterials have been harnessed for infinite applications of organic wastewater treatment [11,12]. The zinc oxide (ZnO) nanoparticles are one of these extraordinary nanomaterials employed for revising the surface characteristics of the hydrophobic membrane’s material [13]. Many pieces of literature claimed that ZnO NMs could impart highly hydrophilic and antifouling features on the target membranes [14,15,16,17]. Based on the nano additives ratio, the performance of the ZnO modified nanocomposite membranes may vary against organic foulants [18].

This work shed the light on an efficient wastewater treatment system that combined an electro-membrane bioreactor (EMBR) and CNTs modified commercial polyurethane sponges as a filter. Herein, both CNTs and polyurethane sponges play significant roles in designing the water purification process. The CNTs were functioning as high-performance electro-catalysts for pollutants degradation (through electron transfer). In this context above, ZnO-modified mixed matrix membranes were fabricated at a dissimilar loading ratio to determine the optimum performance. This CNTs-sponge-MBR (NSMBR) system was designed for removal of NO_3_, PO_4_-P, and Chemical Oxygen Demand (COD), as real wastewater, in order to mitigate membrane fouling. The tests were conducted by playing with operational conditions.

## 2. Experimental

### 2.1. Materials

Polyphenyl sulfone (PPSU-Radel R-5000), with a Mw of 50 KDa, was purchased from Solvay Advanced Polymers (Brussels, Belgium). N-methyl-2-pyrrolidone (NMP) and sodium dodecylbenzene sulfonate (SDBS) were purchased from Sigma–Aldrich (St. Louis, MO, USA). ZnO nanoparticles (10–30 nm diameter) were purchased from SSNano, (Houston, TX, USA). Multi-walled carbon nanotubes have been obtained from Suzhou Jiedi Nano Technology Co., Ltd., (Suzhou, China). The polyurethane sponge was purchased from Shanghai YueXia Industrial Co., Ltd., (Shanghai, China). All chemicals were used as it is without further purification.

### 2.2. Nanocomposite Membrane Preparation

A ZnO-PPSU nanocomposite membrane was prepared via the classical phase separation technique. Briefly, 15 wt.% of PPSU was dissolved in 85 wt.% NMP and stirred overnight until a homogeneous dope solution was achieved. Only one loading weight (1.5 g) of ZnO NPs was added to the casting solution here in the current work. The membrane was cast via an automatic thin-film applicator at a 200-µm clearance gap. Following that, the prepared films were immersed in a deionized water (DI) coagulation bath until complete phase separation is achieved. The membrane was rinsed thoroughly and placed in a glycerol solution to save the membrane’s structure. The amount of ZnO adopted was based on our previous work results where the nanocomposite membrane prepared at 1.5 g has showcased the optimum performance. A more detailed description could be found elsewhere [19].

### 2.3. Fabrication of the CNT Sponge Filter

**Figure 1:**
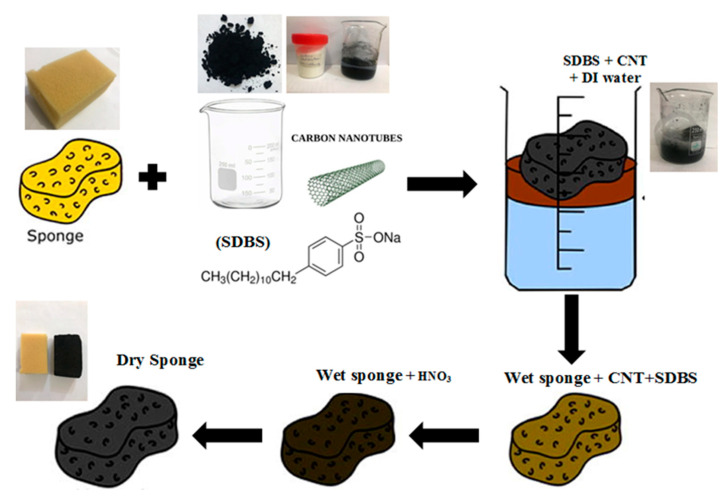
Schematic diagram of the fabrication process.

The conductive sponge has been produced according to a procedure given in a preceding literature [12]. The CNTs ink preparation process was initiated by dispersing 0.3 mg/mL CNTs and 2 mg/mL SDBS in Di water for 15-min. SDBS was employed to encourage homogeneous CNTs dispersion in the aqueous solution and to avoid agglomeration. Later, a piece of polyurethane sponge was immersed in the CNTs suspension and then dried in an oven at 50 °C for 30 min. The high porosity of the sponge encourages the solution’s rapid penetration into the sponge structure [20]. The color change of the sponge sample endowed a convincing evidence for the successful coating process onto the sponge surface. The surface of the sponge became relatively rougher when compared to the uncoated sample. Herein, CNTs could function as a high-performance electro-catalyst for adsorption and oxidation of pollutants due to their excellent electrical conductivity. It could also facilitate the functionalization of polyurethane sponges as a conductive anode. The coated sponge was cut to fit the membrane’s module cell with dimensions of 10 cm × 15 cm × 4 cm. This coating process was repeatedly conducted three times at least to enhance the CNT loading, and, ultimately, the sponge’s electrical conductivity. Thereafter, the prepared CNTs-sponges were treated for 6 h with 4 mol/L HNO_3_ solution at room temperature, aiming to eliminate the remaining surfactant. Finally, the CNT sponges were moved to the NSMBR configuration ready for the tests. The schematic diagram of the fabrication process was depicted in Figure 1 below. It was reported that CNTs–sponge could be sufficiently conductive to function as an electrode in small scale applications. It is also possible to host the conductive CNTs with other support, such as cotton.

### 2.4. Membrane Characterization

Scanning electron microscopy (SEM) has visualized the surface and cross-sectional morphological changes. A TESCAN VEGA3 SB instrument, SEM image (EO-Service, Kohoutovice, Czech Republic). Prior to imaging, using freeze smashing in liquid nitrogen, the membrane cross-sections were prepared while all membranes were sputtered with a thin chromium coat. 

An atomic force microscope (AFM) (model AA3000, Angstrom Advanced Inc., Stoughton, MA, USA) was used for inspections of surface topography of the nano-composite membrane. The membrane pore size and its distribution can be tested by AFM. The most common implementation of this technique uses a cantilever with a sharp tip to scan over the sample surface to get an image at an atomic level. By utilizing the AFM test, van der wall forces and charges of the surface between the tip and the surface of the membrane could be determined. AFM characterization can also give information about the fouling and antifouling behaviour of the membrane since these properties depend on the surface smoothness. Obtained images were processed by using an IMAGER 4.31 statistical program.

A contact angle instrument (CAM 110-O4W, Taiwan) was employed for measurements of the wettability of the PPSU-ZnO membranes. Approximately 4 μL of distilled water was injected on the surface of the membrane and the instrument program captured the contact angle (CA) between the drop and the surface. There were five triplicates taken and average values of CA were presented with a standard deviation.

### 2.5. MBR Experimental Setup

Initially, a real wastewater source obtained from a local domestic wastewater treatment plant (WWTP) has been utilized for the activated sludge-membrane bioreactor (ASMBR) system. All characteristics of the wastewater were summarized in Table 1. The activated sludge was grown in a local WWTP while the biomass was acclimatized to the wastewater for two months to ensure stable conditions before the membrane filtration experiments. The mixed liquor suspended solids (MLSS) concentration, the sludge retention time, and organic loading rate were maintained at 10,000 mg/L, 24-days, and 0.2 g COD/g MLSS-day, respectively. The effective membrane area was 0.000887 m^2^ with a module cell dimension having 10 cm × 15 cm. All experiments were performed under the conditions of −0.4 bar and 25 ± 2 °C.

The second step was divided into two stages, NSMBR and Nano-sponge electro-membrane bioreactor (ENSMBR), respectively.

**Figure 2:**
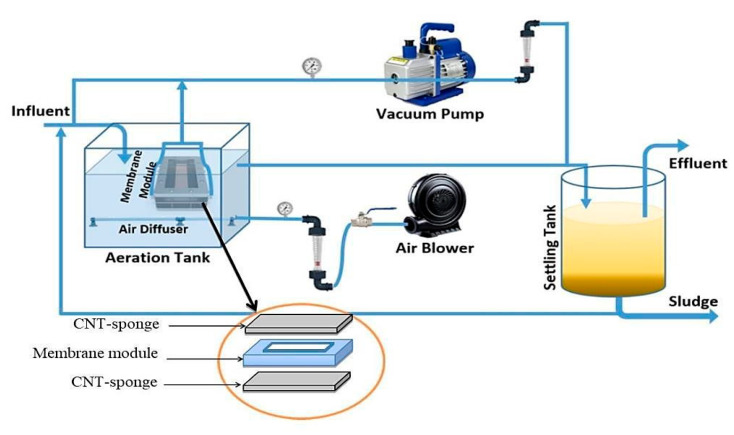
Schematic diagram of the lab-scale AS-MBR system.

**Step one:** The proposed configuration of the ASMBR pilot plant, employed in the current work, comprised a 40-L aeration tank with an effective volume of 32 L. In the schematic diagram of the AS-MBR system (Figure 2), the wastewater was fed into the reactor through a batch feeding to control the feed rate while a suction pump was used to control the effluent flow rate. Air was pumped from the base of the aerobic tank to provide the microorganisms with the necessary oxygen. Moreover, to establish a cross-flow velocity throughout the flat-sheet membrane surfaces, it aimed to diminish MBR fouling. Herein, an intermittent filtration mode was adopted, i.e., 10-min suction and 1-min repose (non-suction).

In step two, a similar two tanks with a working capacity of 40 L and an effective volume of 32 L have been employed for the NSMBR. During the first stage (NSMBR), the test was performed without applying an electric field. The NSMBR configuration consisted of a tank with a membrane module and an aeration mechanism. It should be noted here that the CNTs-sponge was set up to surround the membrane module. Hence, it played as a barrier to contaminants, which reduced the fouling of the membrane.

**Figure 3:**
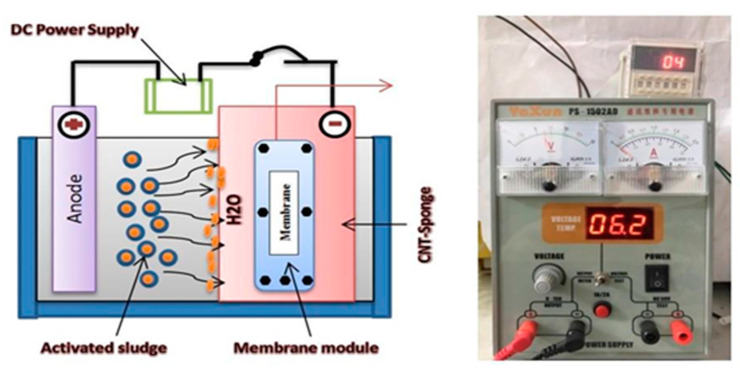
(**Left**) Schematic diagram of carbon nanotubes (CNT)-sponge configuration, (**Right**) DC power supply and timer model.

In the second stage, the ENSMBR configuration consisted of the same components as the NSMBR configuration except for the electrical field application. Herein, two electrodes were employed (anode and cathode). The anode was made of an aluminum rod whereas the cathode was made of lightweight iron mesh around the CNTs-sponge (Figure 3-Left). The electrodes have been connected to an electronic external DC power supply (Yaxun, PS 1502 AD, Guangzhou, China). A timer (Yueqing Landir Electric co., Ltd., model DH48S, Wenzhou, China) was connected to a DC power source to monitor the intermittent DC supply mode (Figure 3-Right).

In the ENSMBR, the aeration system consisted of a porous air diffuser located in the vicinity of the lower center of the electro-bioreactor below the membrane module. It has continuously supplied air to keep the necessary dissolved oxygen (DO) level above 5 mg O_2_/L with the support of an air pump. The supplied air also disturbed the sludge and was intended to reduce membrane fouling [21].

### 2.6. Long-Term Experimental Procedure

The reactor has been operated continuously in two stages. The first stage was carried out for 60 days without wasting the biosolids (SRT = 20 days) while continuing to be supplied with wastewater. Herein, the aeration in the aerobic tank remained continuous. Meanwhile, the ZnO-PPSU membrane was operated at a constant transmembrane pressure. At this stage, no backwashing of the membrane would be performed before the operation of the NSMBR. Meanwhile, during the 90 days of the second stage, the ENSMBR was connected to a power supply in a continuous mode. A constant voltage gradient of 2 V/cm was adopted to be applied at this stage. In order to use the ENSMBR configuration, a preliminary experimental work (stage 2) was carried out to define optimal electro-kinetic conditions. This is essential to define the effective DC field and the intermittence of the exposure period to the DC field. In addition, this is needed to prevent impeding the biological treatment. For this purpose, a series of voltage gradients (1, 2, and 4 V/cm) were applied on a 500-mL activated sludge samples and the optimum value was determined. As a consequence, this value (2 V/cm) was harnessed in the experimental work.

## 3. Results and Discussion

### 3.1. Carbon Nanotube-Sponge Characterization

**Figure 4:**
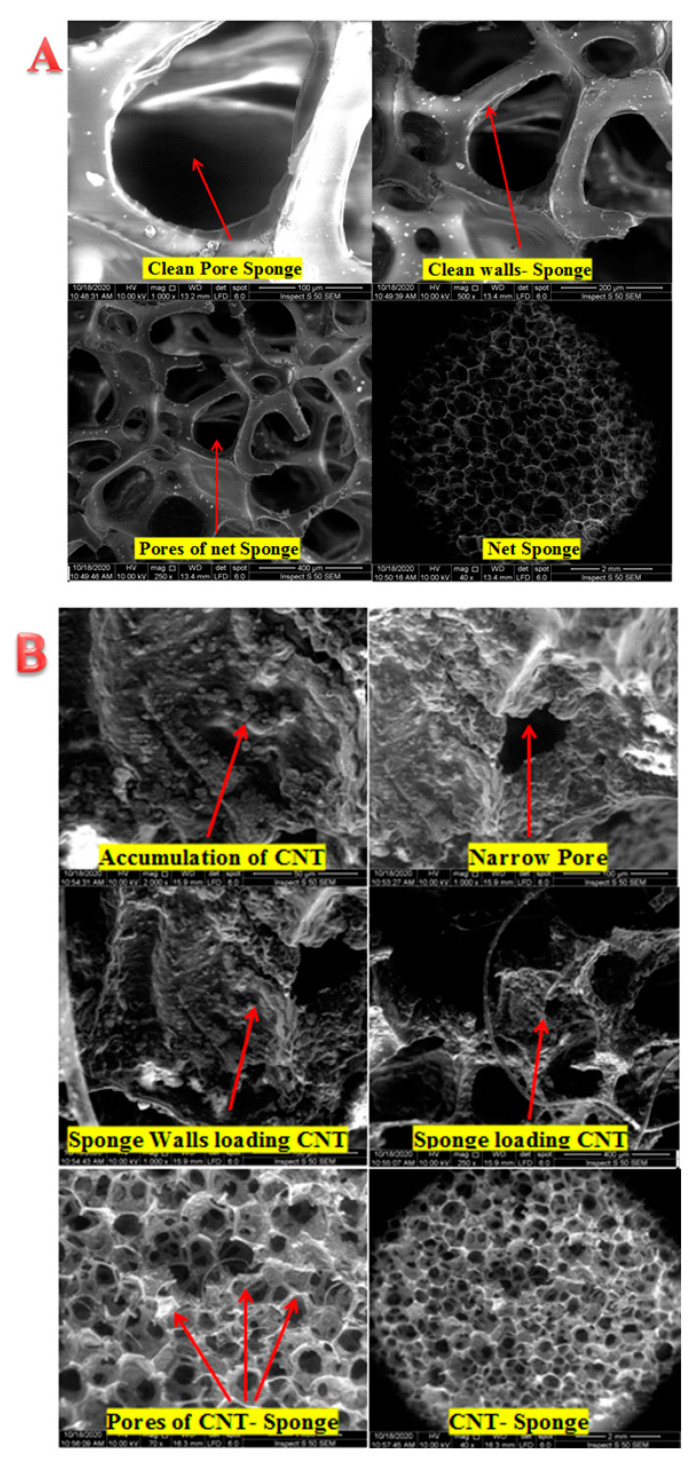
(**A**) SEM images of virgin sponge, (**B**) SEM images of CNTs coated sponge.

MWCNTs was harnessed in this work due to their electro-catalyst characteristics. This promising property could be beneficial for adsorption and oxidation of various pollutants. The coating of MWCNTs into the polyurethane (PU) sponge was visualized by scanning electron microscopy (SEM), as depicted in Figure 4. Figure 4A displayed the high porosity of the PU-Sponge microstructure. As can be seen, there were no CNTs on the walls of the virgin sponge sample that exhibited open-cells with high interconnectivity and are highly desired for the impregnation of CNTs, as reported by [22]. These characteristics are the major reasons standing behind application of the sponge as a template to produce a porous structure. The successful impregnation of CNTs on the sponge surface was illustrated in Figure 4B. This conductive CNTs layer is expected to endow featured characteristics on the ENSMBR system to mitigate fouling. After the coating, the CNT-coated sponge has kept the mechanical characteristics of the uncoated sponge. It has preserved its flexibility and could be bent arbitrarily to any degree with stretchable and compressible structure. These excellent characteristics, e.g., large surface area, high porosity, and high flexibility, make CNT-sponges a favorable candidate as an adsorbent for the pre-concentration of environmental contaminants [23]. The CNTs were found to have inner diameters of 10–20 nm and an external diameter of 30–40 nm along with lengths of tens to hundreds of microns. A clear uniform distribution of the CNTs was observed at the surface of the polyurethane surface, indicating a successful coating process. As depicted in Figure 4B, a thick layer of CNTs was observed at the sponge surface, giving rise to a rougher surface comparing with the virgin sponge surface. Additionally, there was a clear reduction in the pore size of the sponge’s inner structure, and morphology was recognized.

### 3.2. Membrane Surface Characteristics

**Figure 5:**
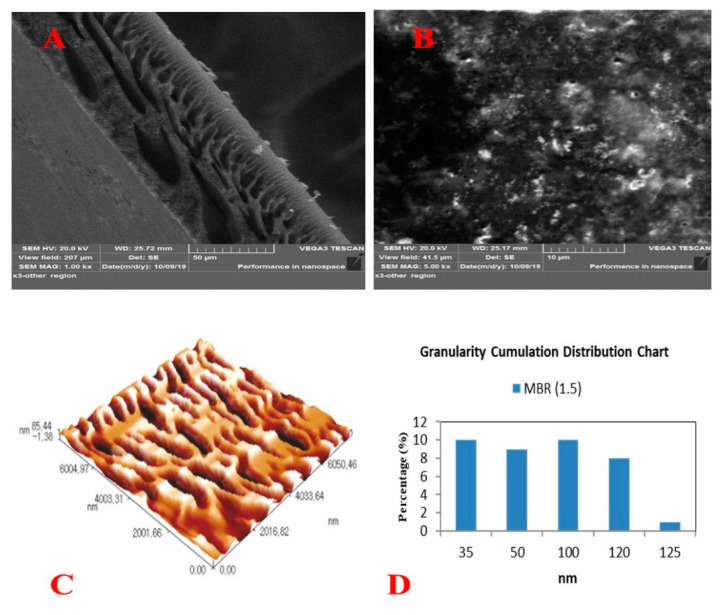
Surface characteristics of the nanocomposite membrane (**A**) SEM cross-section, (**B**) SEM surface, (**C**) AFM, and (**D**) granularity cumulation distribution of pores.

As previously mentioned, the amount of ZnO adopted was based on our previous work results where the nanocomposite membrane prepared at 1.5 g has showcased the optimal performance. The full characteristics of the nanocomposite membranes were depicted and listed in Figure 5 and Table 2, respectively. A typical cross-section structure consisted of two layers: a sponge structure with a big macrovoids layer near the bottom surface and a finger-like structure near the top surface (Figure 5A). This impact of nanoparticles addition was more explicit on the top surface of the membranes. A semi-porous surface with low pore density and clear homogenous spreading of ZnONPs has been observed at the surface (Figure 5B). Surface roughness characteristics were scanned by AFM (Figure 5C). The roughness parameters, Ra and Rms, were found to be 32.5 and 37.6 nm, respectively (Table 2). The mean pore size and cumulative distribution of pores have been illustrated in Figure 5D.

### 3.3. Membranes Performance and Long-Term Operation

The first step of operation was conducted without utilizing the conductive sponge and DC supply (reference stage). As given in our previous work (19), results demonstrated that the prepared nanocomposite membrane using 1.5 g of ZnO NPs has enclosed the longest operation and was chosen for the ASMBR stage. Hence, the entire improvement in the permeate flux due to the addition of 1.5 g ZnO NPs was 450% higher compared with that of control PPSU membrane (not presented results). 

**Figure 6:**
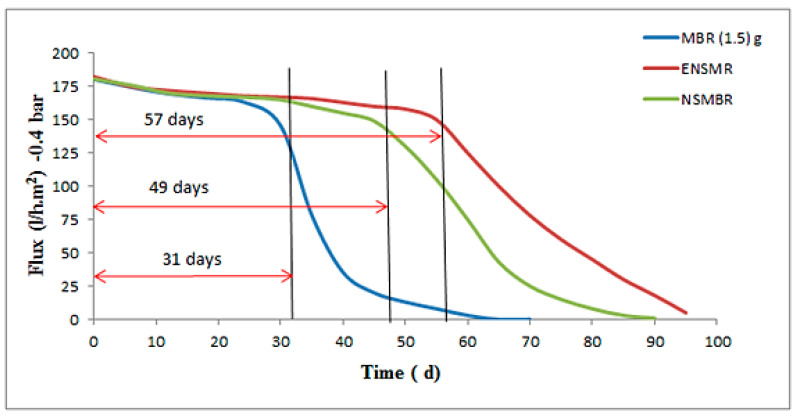
Step II, The effect of the electro field on the lifetime of the PPUS/ZnO NPs membranes in the NSMBR and ENSMBR system.

Figure 6 manifested the long-term performance of the three configurations tested here in this work. During step two, the conductive sponge without applied electrical field was harnessed in NSMBR configuration (Stage I). At this stage, the influence of CNTs-sponge was distinguishable for the biological treatment and antifouling properties of the nanocomposite membrane. The permeate flux before day 30 was almost the same for all systems, see Table 3. The prolonged operation of the membrane was enhanced from 31 days for ASMBR to 49 days for the NSMBR configuration. This influence was imparted by the conductive CNTs-sponge, which enhanced the quality of feed water passed later through the membrane. With this configuration, the membrane was operated until 90 days before the need for cleaning due to the fouling reduction obtained (Figure 6). The CNT-Sponge adsorbed the organic matter and microorganisms’ products. Therefore, the membrane fouling decreased. After that, (2 V) voltage was applied to the CNT sponge anode and the aluminium road cathode to induce the electro-oxidation of the pollutants. The impact of electricity and CNT-sponges on the prolonged operation of nanocomposite membranes prepared from 1.5 g ZnO NPs in ENSMBR (stage II) systems presented the next improvement step for stage I. Application of the CNT-Sponge and the DC with the MLSS solution has initiated electrocoagulation to mitigate fouling of the membrane. It is worth mentioning here that the decline that occurred in the permeate flux of NSMBR was sharp, which is opposite to that of the ENSMBR that deteriorated gradually up to 96 days. In the ENSMBR configuration, the effect of the Nano-sponge with the electric field was prominent on the long-term operation and reached 57 days.

Based on the results mentioned above, it was clear that the electric field had a direct impact on the activated sludge, which is thought to control the repulsion/attraction with the electrodes due to the charge difference. The majority of the sludge in the solution was trapped by the Nano-sponge and diminished the solution viscosity. Both causes reduced the accumulation of the sludge on the membrane and mitigated membrane fouling.

### 3.4. Biological Treatment

#### 3.4.1. COD Removal

**Figure 7:**
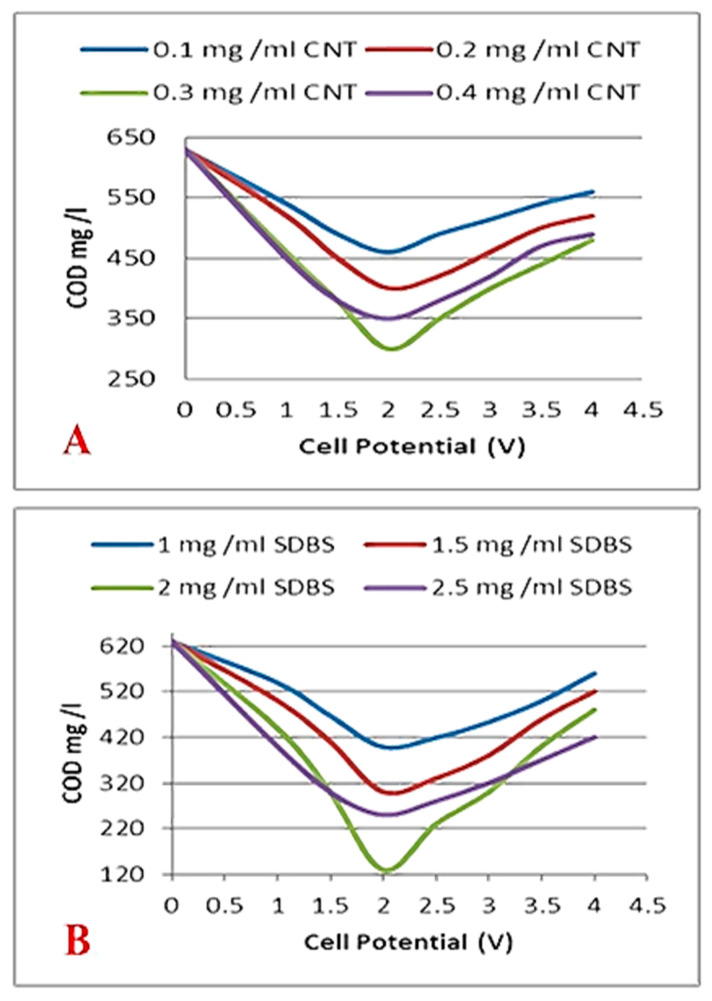
Electro-oxidation COD as a function of (**A**) carbon nanotube (CNT) loading, (**B**) surfactant concentration, and total cell potential.

Figure 7 shows the capability of the CNTs-sponge to degrade the COD concentration (600–800 mg/L). As can be seen, the rate of electro-oxidation of organic matter has been raised by increasing the cell potential from 1.0 V to 2 V. A further increased change in cell potential over 2.0 V induced a reduction in sponge efficiency due to the presence of many side reactions (e.g., water oxidation and/or by-product oxidation). In addition, the blockage of CNTs’ active sites by adsorbed by-products resulted from organic oxidation. For instance, the optimum cell potential of 2 V was sufficiently acceptable following other recorded literature values for the electro-oxidation of organic matters. Here, the CNTs would easily oxidize the organic matter molecule’s dimethylamine group at a fairly low applied potential (e.g., +0.5 V vs. Ag/AgCl) [24]. The load amount of CNTs is considered a significant parameter for removing COD. As shown in Figure 7A, COD removal has increased as the nanotubes loading amount increased. Only 28% of COD could be removed at an imposed cell potential of 2 V if the CNTs loading was 0.1 mg/mL. However, when the CNTs load has increased to 0.3 mg/mL, the removal of COD by electro-oxidation could achieve almost double its value (52%) when compared to 0.1 mg/mL CNTs’ load. The enhanced accessibility of the organic matter molecules into the CNTs’ active centers, at a higher CNTs load, may support the explanation of these results.

Meanwhile, the concentration of the surfactant (SDBS) may also influence the oxidative efficiency of the conductive sponge (Figure 7B). Raising the SDBS concentration could positively participate in the removal of organic matters by electro-oxidation. At 2 V applied cell potential, the oxidation kinetics for the 1.0, 1.5, 2, and 2.5 mg/mL SDBS concentration have showcased 37%, 52%, 80%, and 60%, respectively. Despite its chemical structure, SDBS is an effective surfactant for dispersing MWCNTs in DI water. 

Finally, the removal efficiency of COD in the electro-bioreactor zones has witnessed an enhancement from 75%–90% in stage I to 85%–95% in stage II. This occurred due to the role of the electro-coagulation and CNT-sponge adsorption during stage II. These results appeared to be reasonable and agreed with others reported in the literature [25]. The application of the DC field to the mixed liquor would also diminish the load of the dissolved organic matters onto the membrane fouling due to the repulsion forces. This had been reflected by the good performance of the NSMBR and ENSMBR configurations in stage II where the fouling rate has manifested a significant decrease.

#### 3.4.2. Impacts of MWNTs on Contaminants Removal

Removal of NH_4_-N, NO_3_-N, and PO_4_-P was examined through the ASMBR, NSMBR, and ENSMBR systems, and results were listed in Table 4 below. In the control reactor (step I), the concentration of NH_4_^+^-N and PO_4_-P was not significantly eliminated in the ASMBR because the activated sludge was unable to remove nitrogen and phosphorus compounds as a standalone configuration. At the second step, the NH_4_^+^–N and phosphorus were efficiently eliminated in the NSMBR. This was ascribed to the superior performance of the CNT-sponge in the adopted configuration. The removal efficiencies of total nitrogen (TN) and soluble orthophosphorus (SOP) at the end of the reaction were 70% and >90%, respectively.

Applying the DC field to the mixed liquor had also contributed to the degradation of the dissolved organic matters. Moreover, many organic compounds could be adsorbed by CNTs and, ultimately, led to a reduction in the concentrations of the contaminants.

According to the experimental results, when the dose of MWNTs increased from 0 mg/L to 0.1 mg/mL, there was no substantial difference in the concentration variations of NH_4_^+^–N, NO_3_–N, and NO_2_–N at any time. Besides, there were no adverse effects on the phase of transformation or elimination. However, incorporating 0.3 mg/mL MWNTs has given rise to the highest efficiency for TN (70%) and TP (90.6%) removal.

## 4. Capital Cost

The economic feasibility of the development of the MBR process has been studied in this work to confirm the feasibility of adding PUs, PUs/MWCNTs, and electrical power on the performance of the developed MBR. Table 5 depicts the capital cost of the three different configurations (e.g., MBR, NSMBR, and ENSMBR) according to the several parameters such as energy oxygen required, energy consumption, and cost of the electrodes. The conventional MBR was operated for 31 days while the two developed MBR have a long-term operation of 49 and 57 days for NSMBRs and ENSMBRs, respectively, as shown in Figure 6. The capital cost of the three MBR configurations was evaluated initially for one month. From Table 5, it can be noticed that the capital cost of both NSMBRs and ENMBRs operations recorded a cost reduction of more than 100%.

## 5. Conclusions

Affordable, efficient, and low-energy water purification technologies are highly desirable to handle present-day environmental concerns. Throughout this work, a low-cost methodology was evolved to impart an effective degradation for organic contaminants. This was conducted by harnessing carbon nanotubes as conductive nanomaterials within a polyurethane sponge structure. The nanotubes have been used as a high-potential electro-catalyst for adsorption and oxidation of the pollutant besides functionalization of the sponge to be employed as a conductive anode. In the meantime, the phase inversion was employed to prepare Zinc Oxide PPSU nanocomposite membranes at 1.5 g of loading weight. This additive ratio was selected based on the optimum results obtained in our previous work. A prepared nanocomposite membrane surface was fully characterized by serious experimental tools, e.g., SEM, AFM, contact angle, pore size, and pore size distribution. Results disclosed that the mean pore size was 72.5 nm with a wide pore size distribution that ranged from 35 to 120 nm. Results of hydrophilicity, Ra and Rms were 48 nm, 32.5 nm, and 37.6 nm, respectively. The testing procedure was performed through an ASMBR as a reference and results were compared with those obtained with nanotubes coated sponge–MBR (NSMBR) and nanotubes coated sponge-MBR in the presence of an electrical field (ENSMBR) system. The conductive sponge was attached to the ASMBR system as a pre-treatment to induce the removal of the organic matter and alleviate membrane fouling. Application of the electrical field (2 V) has imparted further enhancement for the treatment process. Results manifested that the long-term operation of ENSMBR was 57 days compared to 31 and 39 days for MBR and NSMBR, respectively. The fouling reduction was 78% compared to the MBR, which showed 60%. Meanwhile, at 0.3 mg/mL, the removal of COD by electro-oxidation could achieve almost double its value (52%) when compared to 0.1 mg/mL CNTs load. Removal of PO_4_ by ENSMBR was almost triple (90.6%) than that obtained by MBR (33.6%). In addition, NO_3_ was eliminated by 81%. These results indicated that application of ENSMBR could have a promising potential when applied for wastewater treatment.

## Figures and Tables

**Table 1:** Characteristics of domestic wastewater.

Pollutant	Influent Concentration (mg/L)	Maximum Allowable Limit (mg/L)
l	450–800	100
NH_3_	150–180	0.02–0.06
NO_3_	25–70	50
P	16–24	3
EC	2393–5102	200–800 μS cm^−1^
TDS	500–860	100
TSS	89–113	20
Pb	0.005	0.05
Cd	0.0025	0.1
pH	7.2–7.4	6.5–8.5

**Table 2:** 1.5 PPSU/ZnO membrane surface characteristics.

Characteristic	Value
Mean Pore Size (nm)	72.57 nm
Granularity Accumulation Distribution of pores (nm)	35–120 nm
Mean Roughness, Ra (nm)	32.5 nm
Rms	37.6 nm
Contact angle	48.67° ± 2.5

**Table 3:** Periods of the membrane life span and the deadline for step II.

Configurations	(Flux) i (L/m^2^ d) = 0 d	(Flux) n (L/m^2^ d) = 30 d	(Flux) n (L/m^2^ d) = 60 d	(Flux) f (L/m^2^ d) = 90 d	Long Term (d)
MBR (1.5 gZnO)	180	150	0.5	0	31
NSMBR	180	167	50	1	49
ENSMBR	180	170	140	50	57

**Table 4:** Nutrient removals in membrane bioreactor (MBR), nanotubes coated sponge–MBR (NSMBR), and electro-membrane bioreactor (ENSMBR).

Experimental Runs and Applied Voltage Gradient (2 V)				
		MBR	NSMBR	NSEMBR
**NH_4_-N (mg/L)**	Influent	31.4 ± 3.1	26.4 ± 2.8	28.5 ± 3
	Effluent	18.7 ± 1.5	11 ± 1.45	6.8 ± 0.6
	**Removal (%)**	**40.4**	**58.3**	**76.14**
**NO_3_-N (mg/L)**	Influent	40.6 ± 3.6	19.3 ± 2.3	21.8 ± 2.6
	Effluent	14.2 ± 0.86	5.8 ± 0.6	4.1 ± 0.54
	**Removal (%)**	**65**	**70**	**81.2**
**PO_4_-P (mg/L)**	Influent	11 ± 1.67	9.4 ± 1.3	11.6 ± 1.2
	Effluent	7.3 ± 0.5	3.1 ± 0.4	1.1 ± 0.2
	**Removal (%)**	**33.6**	**67**	**90.5**

**Table 5:** Capital cost differences among the MBR, NSMBR, and ENSMBR.

Parameter		MBR	NSMBR	ENSMBR
Energy oxygen required (1 Amp/month)		10$ (4 O_2_ mg/L)	10$ (4 O_2_ mg/L)	10$ (4 O_2_ mg/L)
Energy consumption (124 Amp/month) (15 min ON/45 min OFF)		0$	0$	14$/month
Electrodes cost	anode (Sponge)	0$	3$	3$
	cathode	0$	2$	2$
Total cost/month		10$	15$	29$
operation time (d)		31	49	57
Factor operation		31/31 = 1	49/31 = 1.58	57/31 = 1.83
Membrane replace/clogging		yes	No	No
Price membrane replaced		40$	0$	0$
Total cost		50$	−7.7$	−23.1$

0.33$Amp/d. Total cost SMBR = [1 − (Factor operation − 1)] × total cost = (1 − (1.58 − 1)) × 15 = 6.3$.
